# Capsular gene distribution and RAPD typing of *Streptococcus agalactiae* isolated from pregnant women

**DOI:** 10.1186/s13568-024-01671-x

**Published:** 2024-01-28

**Authors:** Mona Zakerifar, Hamid Reza Goli, Hami Kaboosi, Zahra Rahmani, Fatemeh Peyravii Ghadikolaii

**Affiliations:** 1 Department of Microbiology, Ayatollah Amoli Branch, Islamic Azad University, Amol, Iran; 2 Molecular and Cell Biology Research Centre, Faculty of Medicine, Mazandaran University of Medical Sciences, Sari, Iran; 3 Department of Medical Microbiology and Virology, Faculty of Medicine, Mazandaran University of Medical Sciences, Sari, Iran; 4 Department of Obstetrics and Gynecology, Faculty of Medicine, Mazandaran University of Medical Sciences, Sari, Iran; 5 Department of Biology, Qaemshahr Branch, Islamic Azad University, Qaemshahr, Iran

**Keywords:** *Streptococcus agalactiae*, Capsular genes, RAPD typing

## Abstract

*Streptococcus agalactiae* has different virulence factors, from which the capsule has the most significant role in the pathogenesis of this organism. We aimed to investigate the distribution of more prevalent capsular genes among different Random Amplified Polymorphic DNA (RAPD) types of *S. agalactiae* isolated from pregnant women. A total of 106 isolates were collected from 420 vaginal and rectal swabs obtained from pregnant women. The specimens were transferred using Todd Hewitt Broth and were cultured on a blood agar containing antibiotics. The *S. agalactiae* isolates were identified by the standard microbiological and biochemical tests. The genomic DNAs of *S. agalactiae* isolates were extracted using an extraction kit. Then, the PCR method was used to detection of the capsular genes. Moreover, The RAPD PCR was used to genotyping of the isolates. The colonization rate of the pregnant women was 25.23%, and there was a statistically significant correlation between the weeks of gestation and the probability of colonization (p-*value* < 0.05). Also, 31 (29.24%) and 18 (16.98%) pregnant women had a history of abortion and membrane rupture, respectively. In addition, 20 (18.86%), 32 (30.18%), 4 (3.77%), and 6 (5.66%) isolates carried genes encoding capsular types Ia, Ib, III, and V, respectively. None isolates had the type II capsular gene, and other 44 isolates were non-typeable. Nine clones (clusters) of *S. agalactiae* were observed in the present study with 70% similarity, and 53 different types were identified among the isolates. Except for capsular types III and V that belonged to clones 3, 5, 7, and 9, other capsular types were detected in different RAPD types. We found that the capsular types Ib and Ia were predominant among pregnant women in this area, indicating their significance for vaccine designation. Also, our isolates showed a lower genotypic diversity in RAPD typing. This may be due to the same sources of most isolates.

**Supplementary Information:**

The online version contains supplementary material available at 10.1186/s13568-024-01671-x.

## Introduction

*Streptococcus agalactiae* is detected as a vaginal and intestinal microbiota in women causing concerning health associated problems in newborns (do Nascimento et al. 2019; Arabestani et al. 2017; Khademi and Sahebkar 2020). This bacterium is the cause of the early onset disease and late onset disease in neonates delivered by mothers with risk factors, including the delivery before the 37th week of pregnancy, the membrane rupture at least 18 h before delivery, an unexplained fever (≥ 38 °C) during delivery, and a *S. agalactiae* caused a history of invasive disease in a previous infant or urinary tract infection in the current pregnancy (Genovese et al. 2020; Khademi and Sahebkar 2020). Despite the intrapartum antibiotic prophylaxis (IAP) for *S. agalactiae* infections with beta-lactams or clindamycin, a considerable rate of stillbirth and infant death occur annually (do Nascimento et al. 2019; Barros et al. 2023). Screening tests are not routinely performed in Iran (Mosayebi et al. 2017), and prophylaxis is done based on risk factors. There are non-considerable reports from Iran about the rate of neonatal early diseases, but two studies reported two term infants born through vaginal delivery affected with brain abscess and sepsis caused by *S. agalactiae* (Mosayebi et al. 2017; Abdollahi et al. 2011). Approximately 11–35% of the pregnant women are colonized by *S. agalactiae* in different areas (Khademi and Sahebkar 2020; do Nascimento et al. 2019; Alzayer et al. 2023). Similarly, the rate of colonization in non-pregnant healthy adults ranged between 20 and 34% (Alzayer et al. 2023). Babies born to colonized women may get early-onset or late-onset infections, from which 1–3% of them will develop an invasive life-threatening disease (do Nascimento et al. 2019; Khademi and Sahebkar 2020; Jamrozy et al. 2023). Recently, two new (and key) recommendations were reported for the prevention of *S. agalactiae* neonatal infections (NAAT and Encourage 2020; Puopolo et al. 2019). However, screening pregnant women for *S. agalactiae* vaginal and rectal colonization between 35 and 37 weeks of gestation and antibiotic prophylaxis before delivery can reduce the mortality caused by this bacterium (Botelho et al. 2018; do Nascimento et al. 2019). *S. agalactiae* has different virulence factors that are effective in the pathogenesis of this organism. Some significant factors included pore-forming toxins, Christie, Atkins, and Munch-Petersen (CAMP) factor, sialic acid-rich capsular polysaccharide, C5a peptidase, serine proteinase, adhesins, and αC protein (Rajagopal 2009). In addition, the capsule of this organism has the most significant role in pathogenesis of *S. agalactiae* due to the molecular mimicry with the host tissues and the prevention of phagocytosis by the inhibition of the complement factor C3 deposition (Alzayer et al. 2023).

*S. agalactiae* is classified based on capsular polysaccharide antigens, and ten serotypes are currently determined, including Ia, Ib, and II-IX (Botelho et al. 2018; Haimbodi et al. 2021; Huang et al. 2019). Serotypes Ia, II, III, and V are the more prevalent *S. agalactiae* in USA and Europe, causing human diseases (Arabestani et al. 2017). Also, serotypes III, Ia, Ib, II, and V are the most prevalent *S. agalactiae* in neonatal disease, while type III has been associated with severe infections (Arabestani et al. 2017; Barros et al. 2023). On the other hand, according to a meta-analysis of 390 articles, serotypes Ia, Ib, II, III, and V were the most prevalent *S. agalactiae* serotypes colonizing almost 98% of all pregnant women (Huang et al. 2019; Alzayer et al. 2023). An Iranian research reported that serotypes III, V, II, and Ib were the most prevalent *S. agalactiae* isolated from the clinical samples (Emaneini et al. 2014). The prevalence of different capsular serotypes in various isolates of *S. agalactiae* is varied in different parts of the world (Arabestani et al. 2017; Emaneini et al. 2014; Haimbodi et al. 2021; Huang et al. 2019). Because the polysaccharide capsule is one of the most important antigens of this bacterium, determining the capsular serotype seems necessary to determine the suitable candidate for vaccine production in each region (Botelho et al. 2018; do Nascimento et al. 2019; Barros et al. 2023). Although immunological capsular typing is a conventional method for *S. agalactiae*, the polymerase chain reaction-based typing is a considerable method that covers the weaknesses of the previous tests (Arabestani et al. 2017). On the other hand, the genotyping methods, such as Pulsed-field Gel Electrophoresis (PFGE), Multilocus Sequence Typing (MLST), and Randomly Amplified Polymorphic DNA (RAPD) typing, along with capsular typing, can use for better epidemiological study of *S. agalactiae* (Rojo-Bezares et al. 2016). Due to financial limitations and the standardization of RAPD-PCR, we chose this method for molecular typing of our isolates. In this study, we aimed to investigate the capsular and RAPD typing of the *S. agalactiae* isolated from vaginal and rectal specimens of the pregnant women in the present study.

## Materials and methods

### Determination of the sample size and selection of the participants

This study was conducted in Sari, Mazandaran, North of Iran, from March 2021 to September 2021. Based on the statistical parameters of the sample size and according to the following formula, 420 samples were estimated for this study.

$$n\; = \;\frac{{z^2_{{\raise0.7ex\hbox{$\alpha $} \!\mathord{\left/ {\vphantom {\alpha 2}}\right.\kern-0pt}\!\lower0.7ex\hbox{$2$}}} \; \times \;p(1 - P)}}{d^2 }$$

In this formula, n = sample size, p = prevalence of *S. agalactiae* colonization in pregnant women in Iran (p = 12.9%) (Sadeh et al. 2020), d = maximum error rate = 0.032, and Z = value of normal distribution at 95% confidence level (z = 1.96) (Gizachew et al. 2019). The participants were pregnant women at 35 to 37 weeks of gestation who were vaginal or rectal colonized by *S. agalactiae*. The pregnant women were referred to gynecology hospitals and clinics but had no severe diseases and were mentally stable. The participants did not use vaginal creams, sterilizers, and antibiotics in the last two weeks.

### Ethical approval statement and written consent

A questionnaire form was prepared, and the demographic information of the pregnant women and the samples were recorded. A written consent form was prepared, and the essential descriptions concerning voluntary contribution were provided to all participants. The participants completed the consent form before sampling, and were permitted to remove whenever they did not want to continue. This study was conducted according to the Helsinki standards, and all documents was reserved secret. Moreover, our study was accepted by the ethics committee of Mazandaran University of Medical Sciences, and the code of ethics (IR.MAZUMS.REC.1398.418) was assigned to this study.

### Sample collection and identification of bacteria

We used the vaginal and rectal swabs to screen the *S. agalactiae* colonization, as reported previously (Haimbodi et al. 2021; Filkins et al. 2020). The cotton-tipped sterile swabs were used for sampling by a qualified clinician. A Todd Hewitt Broth (Sigma, Germany) containing 8 μg/ml of gentamycin, 15 μg/ml of nalidixic acid, and 5% sheep blood was used to transport the specimens at 2–8 °C (Filkins et al. 2020). After 24 h of incubation of the transport media at 37 °C under 5% CO_2_, 50 μl of the medium was inoculated on 5% sheep blood agar plates (Condalab, Spain), containing the above mentioned antibiotics and was incubated at 37 °C under 5% CO_2_ for 24 h. The *S. agalactiae* identification was confirmed by the observation of the β-hemolytic or non-hemolytic whitish-grey translucent large colonies and the standard microbiological and biochemical tests. These tests included gram staining, catalase, growth on bile esculin agar, hydrolysis of Hippurate, CAMP, and susceptibility to bacitracin (0.04 units) and trimethoprim (1.25 µg)-sulfamethoxazole (23.75 µg) (Tille 2017). *S. agalactiae* ATCC 12403 was used as positive control in this test.

### DNA extraction and polymerase chain reaction

The genomic DNAs of the *S. agalactiae* isolates were extracted using the SinaPure DNA extraction kit (SinaClon, Iran), according to the manufacturer's instructions. Then, the extracted DNAs were electrophoresed on agarose gel (Wizbiosolutions, South Korea), and the optical density of 260/280 nm was determined using a NanoDrop (Thermo Scientific, USA). The different types of capsular polysaccharide encoding genes were detected by PCR using the specific primers shown in Table 1. Amplification of the genes was done in a final volume of 15 μl using 7.5 μl of master mix (Ampliqon, Denmark). Also, 200–600 ng (1–3 µl) of the template DNAs and ten pmol (1 µl) of each primer (Bioneer, South Korea) were used in different reactions of PCRs to detect capsule encoding genes. The reactions were done using a gradient thermocycler (BioRad, USA) in 34 cycles according to the conditions shown in Table 1. The PCR products, along with a DNA marker (Wizbiosolutions), were electrophoresed on a 1.5% agarose gel (Wizbiosolutions) and were observed using a Gel Documentation device (UVITEC Gel Documentation System, Cambridge, UK). We used the control strains containing the capsule encoding genes, prepared from Tehran University of Medical Sciences, as positive controls.

**Table 1:** The primers used for amplification of the capsular genes and the PCR conditions

Genes	Primer sequences 5′ to 3′	PCR product size (bp)	Initial denaturation	Denaturation	Annealing	Extension	Final Extension	References
*cpsIa*	GGTCAGACTGGATTAATGGTATGC	521	95 °C for 5 min	95 °C for 30 s	45 °C for 30 s	72 °C for 35 s	72 °C for 10 min	(Yao et al. 2013)
	GTAGAAATAGCCTATATACGTTGAATGC							
*cpsIb*	TAAACGAGAATGGAATATCACAAACC	770	95 °C for 5 min	95 °C for 30 s	44 °C for 30 s	72 °C for 35 s	72 °C for 10 min	(Yao et al. 2013)
	GAATAAACTTCAATCCCTAAACAATATCG							
*cpsII*	GCTTCAGTAAGTATTGTAAGACGATAG	397	95 °C for 5 min	95 °C for 30 s	55 °C for 30 s	72 °C for 35 s	72 °C for 10 min	(Yao et al. 2013)
	TTCTCTAGGAAATCAAATAATTCTATAGGG							
*cpsIII*	TCCGTACTACAACAGACTCATCC	1826	95 °C for 5 min	95 °C for 30 s	55 °C for 30 s	72 °C for 35 s	72 °C for 10 min	(Yao et al. 2013)
	AGTAACCGTCCATACATTCTATAAGC							
*cpsV*	GAGGCCAATCAGTTGCACGTAA	701	95 °C for 5 min	95 °C for 30 s	54 °C for 30 s	72 °C for 35 s	72 °C for 10 min	(Yao et al. 2013)
	AACCTTCTCCTTCACACACTAATCCT							

### RAPD-PCR typing of the isolates

Other genotyping method performed in the present study was the RAPD analysis using the OPS11 primer (5**′-**AGTCGGGTGG-3**′**), as previously described (Martinez et al. 2000). The RAPD PCR was done by adding 7.5 µl of master mix (Ampliqon), 4 µl of sterile distilled water, 1.5 µl (15 pmol) of primer (Bioneer), and 2 µl (6 ng) of the extracted DNA in a final volume of 15 μl. The PCR was performed by a BioRad thermocycler (USA) under the condition, including an initial denaturation at 94 °C for 5 min, followed by 35 cycles of reactions, comprising a denaturation at 94 °C for 30 s, annealing at 30 °C for 45 s, and an extension at 72 °C for 2 min, followed by a final extension at 72 °C for 10 min. The PCR products, along with a 100 bp plus DNA ladder (Wizbiosolutions), were electrophoresed on a 1.5% agarose gel (Wizbiosolutions), and visualized by the UVITEC Gel Documentation System. To cluster analysis of the isolates, we used the Dice algorithm. Then, a UPGMA type dendrogram was drawn to typing the isolates, and the isolates with a Dice coefficient ≥ 70% were defined as the same RAPD clonal (cluster) type. Also, the isolates were defined as group according to a more similarity (˃ 90%), and the clusters are categorized in the groups (Hanage et al. 2006). *S. agalactiae* ATCC 12403 was used as positive control in this test.

### Statistical analysis

The data were analyzed using Statistical Package for the Social Sciences (SPSS) software version 22. Also, the data comparison was done by Chi-square or Fisher's exact test, and a p-*value* < 0.05 was considered statistically significant.

## Results

### Participants and bacterial isolates

A total of 106 (25.23%) *S. agalactiae* isolates were collected from 420 pregnant women with an average age range of 30.74 ± 5.25 years. Ninety-five (22.61%) and 71 (16.90) pregnant women were vaginal and rectal colonized by *S. agalactiae*, respectively. Also, 59 (55.66%) participants had rectal and vaginal colonization at the same time. The specimens were collected from the participants at 35–37 weeks of gestation who were referred to the Shafa and Nime Shaban hospitals and Moghadam, Royan, and Shahhosseini gynecology clinics. Most participants were housewives, and most were in the 26–30 year age groups. All demographic data related to the participants are shown in Table 2. We detected a significant relationship between the number of positive bacterial cultures and the weeks of gestation (p-*value* < 0.05). Also, 31 (29.24%) and 18 (16.98%) pregnant women had a history of abortion and membrane rupture, respectively.

**Table 2:** The demographic data of the 106 pregnant women included in the present study

Demographic data of the participants	No. (%) of participants	No (%) of isolates containing a capsular type					p-*value*
		Ia	Ib	III	V	Non-typeable	
Age ranges (years-old)							
20–25	17 (16.03)	4 (23.52)	6 (35.29)	0	0	7 (41.17)	0.12
26–30	37 (34.90)	9 (24.32)	9 (24.32)	2 (5.4)	3 (8.1)	14 (37.83)	0.18
31–35	32 (30.18)	4 (12.5)	9 (28.12)	0	3 (9.37)	16 (50)	0.34
36–40	18 (16.98)	3 (16.66)	7 (38.88)	2 (11.11)	0	6 (33.33)	0.38
41–45	2 (1.88)	0	1 (50)	0	0	1 (50)	0.08
Occupation							
Housewife	88 (83.01)	18 (20.45)	28 (31.81)	4 (4.54)	2 (2.27)	38 (43.18)	0.21
Employee	6 (5.66)	2 (33.33)	0	0	0	4 (66.66)	0.11
Teacher	8 (7.54)	0	0	0	4 (50)	4 (50)	0.08
Physician	2 (1.88)	0	2 (100)	0	0	0	0.00
Other	2 (1.88)	0	2 (100)	0	0	0	0.00
Education level							
High school	47 (44.33)	10 (21.27)	14 (29.78)	4 (8.51)	0	23 (48.93)	0.19
Above High school	59 (55.66)	10 (16.94)	18 (30.50)	0	6 (10.16)	25 (42.37)	0.21
Sampling centers							
Shafa	52 (49.05)	10 (19.23)	15 (28.84)	2 (3.84)	4 (7.69)	21 (40.38)	0.22
Moghadam	43 (40.56)	3 (6.97)	15 (34.88)	2 (4.65)	2 (4.65)	21 (48.83)	0.22
Royan	4 (3.77)	1 (25)	2 (50)	0	0	1 (26)	0.09
Nime Shaban	5 (4.71)	4 (80)	0	0	0	1 (20)	0.04
Shahhosseini	2 (1.88)	2 (100)	0	0	0	0	0.00
Outpatient or inpatient							
Inpatient	56 (52.83)	14 (25)	13 (23.21)	2 (3.57)	3 (5.35)	24 (42.85)	0.18
Outpatient	50 (47.16)	6 (12)	19 (38)	2 (4)	3 (6)	20 (40)	0.16
Pregnancy number							
1	61 (57.54)	9 (14.75)	18 (29.50)	2 (3.27)	2 (3.27)	30 (49.18)	0.25
2	18 (16.98)	1 (5.55)	6 (33.33)	0	2 (11.11)	9 (50)	0.25
3	19 (17.92)	8 (42.10)	6 (31.57)	0	2 (10.52)	3 (15.78)	0.07
4	6 (5.66)	2 (33.33)	0	2 (33.33)	0	2 (33.33)	0.09
5	2 (1.88)	0	2 (100)	0	0	0	0.00
Pregnancy week							
35th	12 (11.32)	1 (8.33)	4 (33.33)	0	0	7 (58.33)	0.23
36th	26 (24.52)	9 (34.61)	6 (23.07)	2 (7.69)	0	9 (34.61)	0.09
37th	68 (64.15)	10 (14.70)	22 (32.35)	2 (2.94)	6 (8.82)	28 (41.17)	0.12
Abortion number							
0	75 (70.75)	15 (20)	23 (30.66)	2 (2.66)	4 (5.33)	31 (41.33)	0.12
1	19 (17.92)	1 (5.26)	5 (26.31)	0	2 (10.52)	11 (57.89)	0.23
2	10 (9.43)	4 (40)	2 (20)	2 (20)	0	2 (20)	0.04
3	0	0	0	0	0	0	–
4	2 (1.88)	0	2 (100)	0	0	0	0.00
Membrane rupture number							
0	88 (83.01)	18 (20.45)	28 (31.81)	2 (2.27)	6 (6.81)	34 (38.63)	0.28
1	18 (16.98)	2 (11.11)	4 (22.22)	2 (11.11)	0	10 (55.55)	0.19

### Detection of capsule encoding genes

Among 106 *S. agalactiae* isolates collected in this study, 20 (18.86%), 32 (30.18%), 4 (3.77%), and 6 (5.66%) isolates carried genes encoding capsular types Ia, Ib, III, and V, respectively. However, no isolates had the type II capsular gene. Other 44 isolates were non-typeable by the primers used in this study. The relationship between the capsular types of *S. agalactiae* isolates and demographic data is shown in Table 2. About 50% of the isolates with capsular type Ia were isolated from Shafa hospital, and the remaining were isolated from other centers (p = 0.0).

Most capsular types were not observed in the 41 to 45 year age group. A significant relationship was observed between the presence of capsular genes and the occupation of pregnant women, so most of the isolates with the studied genes were obtained from housewives (p < 0.05). In addition, not only 50% of the isolates with capsular gene *V* were identified in teachers, but also all six isolates carrying this gene were observed in the above high school group. No significant correlation was found between the outpatient or inpatient status of pregnant women, the number of pregnancies, membrane rupture, or the number of abortions, and the presence of capsule genes in this study. However, as the pregnancy week of the studied women increased, the probability of the investigated capsule genes identification was also increased (p < 0.05).

### RAPD typing of the isolates

**Fig. 1:**
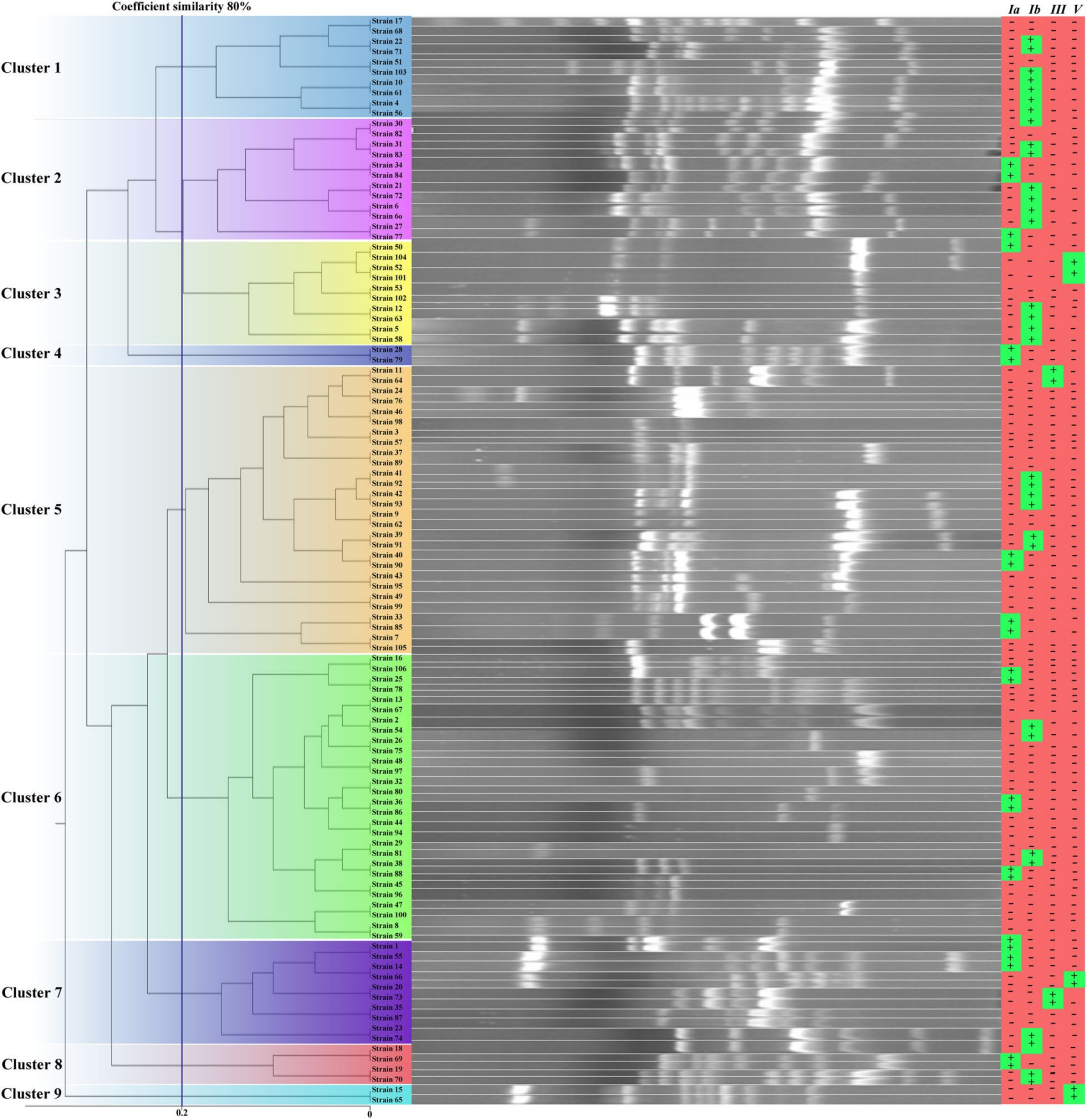
RAPD typing and capsular distribution of *S. agalactiae* isolates collected from pregnant women in this study. The blots are cropped from the original electrophoresis gel. The blots with the same patterns are placed together. Also, the original gels with full-length blots are attached as Additional file 1

We detected 1 to 11 DNA fragments sizing from 180 to 4500 bp in RAPD typing of the 106 *S. agalactiae* isolates (Fig. 1), as previously reported by our team (Zakerifar et al. 2023). There was five groups with a 70% similarity in this study, from which group 1 had four clones (1–4), while group 2 was contained two clones (5 and 6). However, nine clones of *S. agalactiae* were observed in the present study with 70% similarity, and 53 different types were identified among the isolates. Also, the prevalence of capsule encoding genes in *S. agalactiae* clones is shown in the Table 3. Except for capsular types III and V that belonged to clones 3, 5, 7, and 9, other capsular types were detected in different RAPD types (Table 3).

**Table 3:** Prevalence of capsular genes in different clones of *S. agalactiae*

*S. agalactiae* clones (No. of isolates)	The number (%) of isolates carrying capsular genes				*Non-typeable*
	*Ia*	*Ib*	*III*	*V*	
1 (n = 10)	0	6 (60)	0	0	4 (40)
2 (n = 12)	2 (16.66)	8 (66.66)	0	0	2 (16.66)
3 (n = 10)	2 (20)	4 (40)	0	2 (20)	2 (20)
4 (n = 2)	2 (100)	0	0	0	0
5 (n = 28)	4 (14.28)	6 (21.42)	2 (7.14)	0	16 (57.14)
6 (n = 28)	6 (21.42)	4 (14.28)	0	0	18 (64.28)
7 (n = 10)	2 (20)	2 (20)	2 (20)	2 (20)	2 (20)
8 (n = 4)	2 (50)	2 (50)	0	0	0
9 (n = 2)	0	0	0	2 (100)	0

## Discussion

*Streptococcus agalactiae* is the cause of neonatal sepsis, pneumonia, and meningitis, while sepsis is one of the leading causes of death in infants in developing and developed countries (Botelho et al. 2018; Chang et al. 2010). It is estimated that 10–30% of pregnant women are colonized by this bacteria in their vagina or rectum and can vertically transmit this organism to their neonates (do Nascimento et al. 2019; Khademi and Sahebkar 2020). Different prevalence rates of colonization with *S. agalactiae* (1.8% to 27.7%) were reported in Iran (Sadeh et al. 2020). Using the vaginal and rectal culture method, 25.23% of our participants were colonized by *S. agalactiae*, while the vaginal colonization was more than the rectal. A Brazilian research in 2019 reported that 4.2–28.4% of pregnant women were colonized by *S. agalactiae* in the last ten years (do Nascimento et al. 2019). Moreover, the prevalence of *S. agalactiae* in other countries shows different statistics. The prevalence rate in the USA, Thailand, Poland, France, and India were 27.2%, 12.9%, 17.2%, 16.7%, and 2.3%, respectively (Bland et al. 2000; Tor-Udom et al. 2006; Brzychczy-Włoch et al. 2008; Dahan-Saal et al. 2011; Sharmila et al. 2011). The reason for the variety of results could be attributed to differences in age, culture method, inherent differences in populations, and more use of antibiotics in some populations, as well as inadequate skills of personnel in diagnosis.

On the other hand, the sialic acid-rich capsule in *S. agalactiae* is a significant virulence factor effective in pathogenesis (Arabestani et al. 2017). Thus, the investigation of the capsular types in *S. agalactiae* isolates in different areas seems to be significant for the development of a vaccine preventing the intrapartum transport of the bacterium. We aimed to detect the five predominant capsular types (Ia, Ib, II, III, and V) in *S. agalactiae* isolated from pregnant women in Mazandaran, north of Iran. We reported that 62 (58.49%) *S. agalactiae* isolates were typeable by PCR method, while 44 (41.50%) isolates were non-typeable or had other capsular types. A previous study on *S. agalactiae* isolates, collected from pregnant women and clinical settings, used three PCR methods for capsular typing (Yao et al. 2013). They showed that some serotypes did not recognize by PCR tests, and some of them misidentified as other serotypes due to large insertions or deletions affecting the *cps* gene cluster (Yao et al. 2013). Thus, the non-typeable isolates in the present study may be affected by these gene mutations or contained other *cps* genes. The most prevalent capsular genes in this study was related to the *Ib* gene (30.18%), while the *Ia*, *V*, and *III* capsular genes were identified in 18.86%, 5.66%, and 3.77% of the isolates, and none of the isolates carried the type *II* capsule. Unlike our study, Arabestani et al. reported that type *III* was the predominant capsular type (56.5%) in *S. agalactiae* isolated from pregnant women in Hamedan, Iran, followed by types *V* (17.7%), *II* (11.3%), *Ia* (8.1%), and *Ib* (3.2%) (Arabestani et al. 2017). Also, Jannati et al. from Ardabil showed that 19.6%, 12.5%, 10.7%, 8.9%, and 7.1% of their isolates belonged to the V, II, III, Ib, and Ia capsular types by serotyping method, respectively, while 7.1% of their isolates were non-typeable (Jannati et al. 2012). Another Iranian research from Tabriz reported that capsular type V was the most prevalent (19.5%) in pregnant women and neonates, followed by Ia (17.6%), II (14.2%), Ib (13.4%), and III (9.5%), while 17.6% of their isolates were non-typeable by serotyping method (Nahaei et al. 2007). Types *III* and *V* were the most prevalent capsular genes in other Iranian studies (Arabestani et al. 2017). However, the prevalence of capsular genes in our research was different with other Iranian studies. This result indicates that the vaccine development policy is varied in our region but needs more investigation by other primers specific for other capsular types. Although all above mentioned Iranian studies identified capsular serotype II in their study, this serotype was not observed in our isolates. Although we used the standard strain carrying this gene to check the presence of the gene encoding this capsular serotype, but this absence may be due to a work error or the inability of the primer used in our study to identify this gene. This lack of identification can also be due to the mutation in the relevant gene (Yao et al. 2013).

Another study conducted in Saudi Arabia on 140 pregnant women and 95 infected adults showed that 25% of the isolates were harboring the genes encoding the serotypes III and V were, and 16.18%, 13.24%, 9.31%, and 8.82% of the isolates belonged to the serotypes II, Ia, VI, and Ib, respectively, while five (2.45%) isolates were non-typeable (Alzayer et al. 2023). European, American, Asian, and African studies reported different prevalence rates of capsular types. Previous research conducted in the USA showed that types III, V, and Ia were the most prevalent capsular serotypes (Hawkins et al. 2017). In addition, a Brazilian research on 124 *S. agalactiae* isolates collected from pregnant women showed that the Ia encoding gene was most frequent type (33.0%) during the whole period (2002–2018), followed by II (25.8%), V (21.8%), Ib (8.9%), III (8.9%), and IV (1.6%). They found a variation in circulation of the capsular types, specially V, over time (Barros et al. 2023). Other research conducted on 535 *S. agalactiae* isolates collected from pregnant women in London exhibited a that among nine serotypes identified, the most prevalent serotype was III (26%), followed by V (21%), II (19%) and Ia (19%), while the serotypes Ib, IV, VI, VII, and IX had a less than 10% prevalence (Jamrozy et al. 2023). Also, another Iranian study carried out on 60 *S. agalactiae* isolates collected from women with urinary tract infection (UTI) detected the capsular serotype II as the most prevalent (66.7%), while 21.7%, 3.3%, and 1.7% of the isolates were carrying the Ib, Ia, and III capsular encoding genes, and 6.6% of the isolates were non-typeable (Gharabeigi et al. 2023). According to a review by Huang et al. from China, serotype III has a lower prevalence in South American, Asian, and Western African countries, while serotype V is the most prevalent in these regions (Huang et al. 2019). However, a Chines research showed that 41.8%, 21.4%, 14.9%, and 11.9% of the *S. agalactiae* isolates collected from pregnant women contained the III, Ia, V, and Ib capsular types encoding genes, respectively (Lu et al. 2014). Another Chinese research detected a similar result to the present study in terms of capsular gene prevalence, from which 36.7%, 28.3%, and 18.3% of their isolates contained the *Ib*, *Ia*, and *III* genes, respectively (Su et al. 2016). Also, a Brazilian research reported that 37.3%, 11.2%, 19.9%, 6.8%, and 9.2% of their *S. agalactiae* isolates contained the *Ia*, *Ib*, *II*, *III*, and *V* capsular genes, respectively (Botelho et al. 2018). Geographical differences may attribute to the differences in the prevalence of the capsular genes in different areas. The combination of PCR analysis and serotyping may have improved the classification of *S. agalactiae* capsular types.

For molecular typing of *S. agalactiae* isolates, we used the RAPD-PCR method using a primer previously reported (Martinez et al. 2000). We first selected 4 different primers from previous studies and performed the PCR test with each of them alone and in different combinations with each other, but when using the OPS11 primer alone, more electrophoretic bands were observed. Thus, this primer was chosen to perform the RAPD-PCR test. We detected nine clones and 53 different RAPD types of *S. agalactiae* with 70% similarity in the present study. Also, Jamorsy et al. detected nine clone of *S. agalactiae* among 535 isolates collected from pregnant women in London (Jamrozy et al. 2023). A Spanish research that used the PFGE method for typing *S. agalactiae* isolates, were detected 65 different patterns (Rojo-Bezares et al. 2016). We found that all strains with capsular types III and V belonged to the RAPD clones 3, 5, 7, and 9, while other capsular types were distributed among different RAPD types. However, the above-mentioned Spanish research reported that 33.8% and 24.1% of their isolates with different PFGE patterns belonged to capsular types III and V (Rojo-Bezares et al. 2016). As reported by Rojo-Bezares et al., common typing clones have a strong correspondence with capsular serotypes (Rojo-Bezares et al. 2016), but we did not observe this issue except for the types III and V. According to the research conducted by Chatellier et al., the combination of RAPD typing and serotyping is the simplest way for distinguishing the *S. agalactiae* isolates (Chatellier et al. 1997). We used the combination of RAPD and capsular gene typing and found that clone 1 just contained six isolates with capsular type Ib and clone 4 just contained two isolates with Ia capsular type, while clone 9 just contained two isolates with capsular type V. Other clusters in the present study had different isolates in terms of capsule type. Toresani et al. found 16 RAPD profiles among 21 *S. agalactiae* isolates collected from 17 women in Argentina (Toresani et al. 2001). Also, Chatellier et al. detected 71 RAPD types among 54 *S. agalactiae* isolates collected from cerebrospinal fluids of neonates (Chatellier et al. 1997). This genotypic diversity was similar to the research conducted by El Aila et al. in Belgium on 36 *S. agalactiae* isolates collected from pregnant women (El Aila et al. 2009). We detected 53 different RAPD types among 106 *S. agalactiae* isolates in the present study. These results indicate the lower diversity of our isolates compared to the above mentioned studies. However, the capsular typing in the present study was not suitable method for genotyping of the *S. agalactiae* isolates may due to the limitation of the primers used. Thus, we should investigate other capsular genes in our isolates in the future. Amal et al. found 13 different groups among 181 *S. agalactiae* isolates collected from diseased fish, from which six groups were composed of only one strain, while group 4 was contained 70 strains (Amal et al. 2013). We detected five groups in the present study, from which 34 isolates were composed in group 1, and 56 isolates belonged to the group 2, while other 16 isolates were divided in three groups. Our study revealed a 25.23% prevalence of *S. agalactiae* colonization in pregnant women, which was considerable and similar to other studies conducted in Iran and other countries worldwide. The rate of *S. agalactiae* isolation from vaginal swabs was more than the rectal swabs in pregnant women. We found that the capsular types Ib and Ia were predominant among pregnant women in this area. This indicates the significance of these types for development of vaccine designation, especially for this area. On the other hand, our isolates showed a lower genotypic diversity in RAPD typing. This may be due to the same sources of most isolates. However, we found that the capsular typing by PCR method need to the investigation of all capsular genes identified in *S. agalactiae* isolates.

## Limitations

All primers for GBS serotyping were not used. Also, the limitation of the genotyping method was the use of one primer.

### Supplementary Information

**Additional file 1**: **Figure**. Full-length blots of the gel electrophoresis with membranes.

## Data Availability

All data generated or analyzed during this study are included in this published article.

## Abbreviations

RAPD: Randomly amplified polymorphic DNA
DNA: Deoxy ribonucleic acid
CAMP: Christie, Atkins, Munch, and Peterson
PFGE: Pulsed-field Gel
Electrophoresis, MLST: Multilocus Sequence Typing
PCR: Polymerase chain reaction
SPSS: Statistical Package for the Social Sciences
EOD: Early-onset disease
LOD: Late-onset disease

## References

[CR1] Abdollahi A, Moradi-Tabriz A, MehdipourAghabagher A (2011). Group B streptococcal sepsis in a newborn: a case report. Iran j Pathol.

[CR2] Alzayer M, Alkhulaifi MM, Alyami A, Aldosary M, Alageel A, Garaween G, Shibl A, Al-Hamad AM, Doumith M (2023). Molecular typing and antimicrobial resistance of group B Streptococcus clinical isolates in Saudi Arabia. J Glob Antimicrob Resist.

[CR3] Amal M, Zamri-Saad M, Siti-Zahrah A, Zulkafli A, Nur-Nazifah M (2013). Molecular characterization of S treptococcus agalactiae strains isolated from fishes in M alaysia. J Appl Microbiol.

[CR4] Arabestani MR, Mousavi SM, Nasaj M (2017). Genotyping of clinical *Streptococcus **agalactiae* strains based on molecular serotype of capsular (cps) gene cluster sequences using polymerase chain reaction. Archives Clin Infect Dis.

[CR5] Barros RR, Alves KB, Luiz FBO, Ferreira DG (2023). Prevalence of *Streptococcus **agalactiae* capsular types among pregnant women in Rio de Janeiro and the impact of a capsular based vaccine. Brazilian J Pharm Sci.

[CR6] Bland ML, Vermillion ST, Soper DE (2000). Late third-trimester treatment of rectovaginal group B streptococci with benzathine penicillin G. Am J Obstet Gynecol.

[CR7] Botelho ACN, Oliveira JG, Damasco AP, Santos KT, Ferreira AFM, Rocha GT, Marinho PS, Bornia RB, Pinto TC, Americo MA (2018). *Streptococcus **agalactiae* carriage among pregnant women living in Rio de Janeiro, Brazil, over a period of 8 years. PLoS ONE.

[CR8] Brzychczy-Włoch M, Strus M, Pawlik D, Machlarz H, Gosiewski T, Drzewiecki A, Rytlewski K, Lauterbach R, Heczko PB (2008). Increasing *Streptococcus **agalactiae* colonization of pregnant women and newborns in south-eastern region of Poland. Med Dosw Mikrobiol.

[CR9] Chang H, Shen X, Fu Z, Liu L, Shen Y, Liu X, Yu S, Yao K, Zhao C, Yang Y (2010). Antibiotic resistance and molecular analysis of Streptococcus pyogenes isolated from healthy schoolchildren in China. Scand J Infect Dis.

[CR10] Chatellier S, Ramanantsoa C, Harriau P, Rolland K, Rosenau A, Quentin R (1997). Characterization of *Streptococcus **agalactiae* strains by randomly amplified polymorphic DNA analysis. J Clin Microbiol.

[CR11] Dahan-Saal J, Gérardin P, Robillard P, Barau G, Bouveret A, Picot S, Fianu A, Boukerrou M (2011). Determinants of group B streptococcus maternal colonization and factors related to its vertical perinatal transmission: case-control study. Gynecol Obstetri Fert.

[CR12] do Nascimento CS, Dos Santos NF, Ferreira RC, Taddei CR (2019). *Streptococcus **agalactiae* in pregnant women in Brazil: prevalence, serotypes, and antibiotic resistance. Brazilian J Microbio.

[CR13] el Aila NA, Tency I, Claeys G, Saerens B, de Backer E, Temmerman M, Verhelst R, Vaneechoutte M (2009). Genotyping of *Streptococcus **agalactiae* (group B streptococci) isolated from vaginal and rectal swabs of women at 35–37 weeks of pregnancy. BMC Infect Dis.

[CR14] Emaneini M, Mirsalehian A, Beigvierdi R, Fooladi AAI, Asadi F, Jabalameli F, Taherikalani M (2014). High incidence of macrolide and tetracycline resistance among *Streptococcus **agalactiae* strains isolated from clinical samples in Tehran. Iran Maedica.

[CR15] Filkins L, Hauser JR, Robinson-Dunn B, Tibbetts R, Boyanton BL, Revell P (2020). Guidelines for the detection and identification of group B streptococcus. Am Soc Microbiol. https://asm.org/ASM/media/Policy-and-Advocacy/images/ASM-GBS-guideline-031020.pdf. 10.1128/JCM.01230-20 PMC7771461 33115849

[CR16] Genovese C, D’Angeli F, di Salvatore V, Tempera G, Nicolosi D (2020). *Streptococcus **agalactiae* in pregnant women: serotype and antimicrobial susceptibility patterns over five years in Eastern Sicily (Italy). Eur J Clin Microbiol Infect Dis.

[CR17] Gharabeigi N, Bafroee AST, Amini K (2023). Molecular serotyping and antibiotic resistance profile of group B Streptococcus strains isolated from iranian pregnant women with urinary tract infection. Iranian J Med Sci.

[CR18] Gizachew M, Tiruneh M, Moges F, Adefris M, Tigabu Z, Tessema B (2019). *Streptococcus **agalactiae* from Ethiopian pregnant women; prevalence, associated factors and antimicrobial resistance: alarming for prophylaxis. Ann Clin Microbiol Antimicrob.

[CR19] Haimbodi EL, Mukesi M, Moyo SR (2021). Prevalence and molecular characterization of group B streptococcus in pregnant women from hospitals in Ohangwena and Oshikoto regions of Namibia. BMC Microbiol.

[CR20] Hanage WP, Fraser C, Spratt BG (2006). Sequences, sequence clusters and bacterial species. Philosoph Transact Roy Soc B Bio Sci.

[CR21] Hawkins PA, Law CS, Metcalf BJ, Chochua S, Jackson DM, Westblade LF, Jerris R, Beall BW, McGee L (2017). Cross-resistance to lincosamides, streptogramins A and pleuromutilins in *Streptococcus **agalactiae* isolates from the USA. J Antimicrob Chemother.

[CR22] Huang J, Lin X-Z, Zhu Y, Chen C (2019). Epidemiology of group B streptococcal infection in pregnant women and diseased infants in mainland China. Pediatr Neonatol.

[CR23] Jamrozy D, Gopal Rao G, Feltwell T, Lamagni T, Khanna P, Efstratiou A, Parkhill J, Bentley SD (2023). Population genetics of group B Streptococcus from maternal carriage in an ethnically diverse community in London. Front Microbio.

[CR24] Jannati E, Roshani M, Arzanlou M, Habibzadeh S, Rahimi G, Shapuri R (2012). Capsular serotype and antibiotic resistance of group B streptococci isolated from pregnant women in Ardabil. Iran Iranian J Microbio.

[CR25] Khademi F, Sahebkar A (2020). Group B streptococcus drug resistance in pregnant women in Iran: a meta-analysis. Taiwan J Obstet Gynecol.

[CR26] Lu B, Li D, Cui Y, Sui W, Huang L, Lu X (2014). Epidemiology of Group B streptococcus isolated from pregnant women in Beijing, China. Clin Microbiol Infect.

[CR27] Martinez G, Harel J, Higgins R, Lacouture S, Daignault D, Gottschalk M (2000). Characterization of *Streptococcus **agalactiae* isolates of bovine and human origin by randomly amplified polymorphic DNA analysis. J Clin Microbiol.

[CR28] Mosayebi Z, Mohammadi M, Mohammadi M, Jafari A, Movahedian AH (2017). Early-onset meningitis with group B Streptococcus: the case of a female neonate with brain abscess in Iran. J Comprehen Pediat.

[CR29] Naat G, Encourage P (2020) Prevention of group B streptococcal early-onset disease in newborns. 10.1542/peds.2019-1882 31285394

[CR30] Nahaei MR, Ghandchilar N, Bilan N, Ghahramani P (2007). Maternal carriage and neonatal colonization of *Streptococcus **agalactiae* in Tabriz, Northwest Iran. Iranian J Med Sci.

[CR31] Puopolo KM, Lynfield R, Cummings JJ, Hand I, Adams-Chapman I, Poindexter B, Stewart DL, Aucott SW, Goldsmith JP, Mowitz M (2019). Management of infants at risk for group B streptococcal disease. Pediatrics.

[CR32] Rajagopal L (2009) Understanding the regulation of Group B Streptococcal virulence factors. 10.2217/17460913.4.2.201 PMC2691590 19257847

[CR33] Rojo-Bezares B, Azcona-Gutiérrez J, Martin C, Jareño M, Torres C, Sáenz Y (2016). *Streptococcus **agalactiae* from pregnant women: antibiotic and heavy-metal resistance mechanisms and molecular typing. Epidemiol Infect.

[CR34] Sadeh M, Salehi-Abargouei A, Azartoos N, Mirzaei F, Khalili MB (2020). Distribution of *Streptococcus agalactiae* among Iranian women from 1992 to 2018: a systematic review and meta-analysis. Jundishapur J Microbio. 13.

[CR35] Sharmila V, Joseph NM, Babu TA, Chaturvedula L, Sistla S (2011). Genital tract group B streptococcal colonization in pregnant women: a South Indian perspective. J Infect Develop Count.

[CR36] Su J, Wu W, Ding L, Huang J, Wang S, Zhang J (2016). Drug resistance, serotypes, virulence-associated genes and genotypes of infection or colonization of group B Streptococcus in perinatal pregnant women. Chin J Obstet Gynecol Pediatr.

[CR37] Tille MP (2017). Bailey & scott’s diagnostic microbiology.

[CR38] Toresani I, Limansky A, Bogado I, Guardati MC, Viale A, Sutich EG (2001). Phenotypic and genotypic study of *Streptococcus **agalactiae* in vagina of pregnant women in Argentina. Med Buenos Aires.

[CR39] Tor-Udom S, Tor-Udom P, Hiriote W (2006). The prevalence of *Streptococcus **agalactiae* (group B) colonization in pregnant women at Thammasat Hospital. J Med Assoc Thailand.

[CR40] Yao K, Poulsen K, Maione D, Rinaudo CD, Baldassarri L, Telford JL, Sørensen UB, Kilian M, Group, D. S (2013). Capsular gene typing of *Streptococcus **agalactiae* compared to serotyping by latex agglutination. J Clin Microbio.

[CR41] Zakerifar M, Kaboosi H, Goli HR, Rahmani Z, PeyraviiGhadikolaii F (2023). Antibiotic resistance genes and molecular typing of *Streptococcus **agalactiae* isolated from pregnant women. BMC Pregn Childbirth.

