# Supplementary material for: Capsular gene distribution and RAPD typing of Streptococcus agalactiae isolated from pregnant women
Source: AMB Express. 2024 Jan 28;14:13. doi: 10.1186/s13568-024-01671-x (PMC10822826; doi:10.1186/s13568-024-01671-x)
Supplement: Supplementary file 1 — Additional file 1: Figure. Full-length blots of the gel electrophoresis with membranes. [file 13568_2024_1671_MOESM1_ESM.docx]

**Additional file 1: Figure.** Full-length blots of the gel electrophoresis with membranes


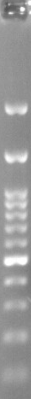

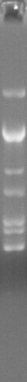

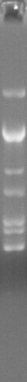

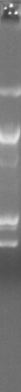

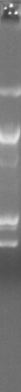

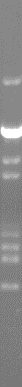

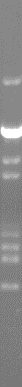

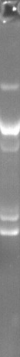

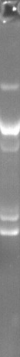

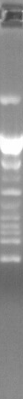

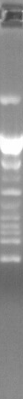

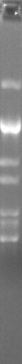

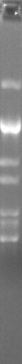

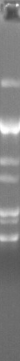

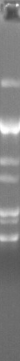

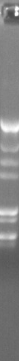

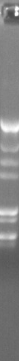

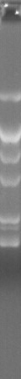

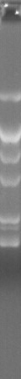

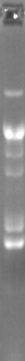

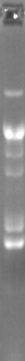

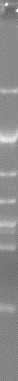

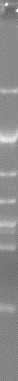

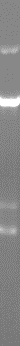

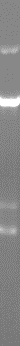

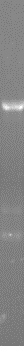

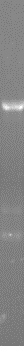

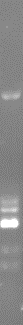

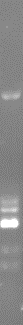

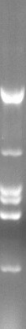

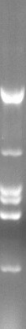

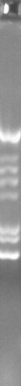

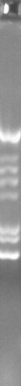

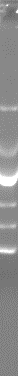

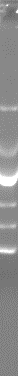

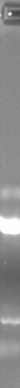

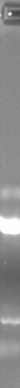

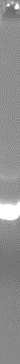

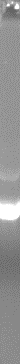

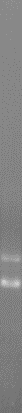

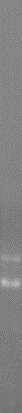

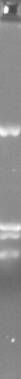

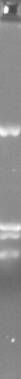

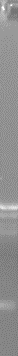

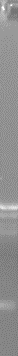

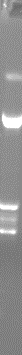

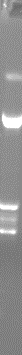

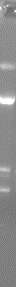

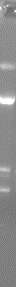

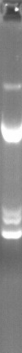

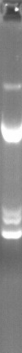

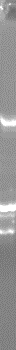

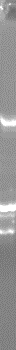

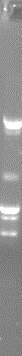

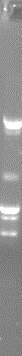

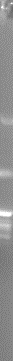

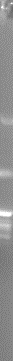

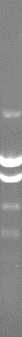

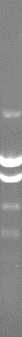

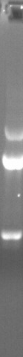

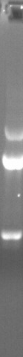

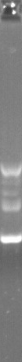

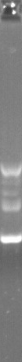

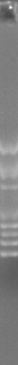

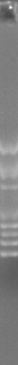

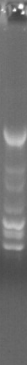

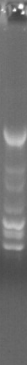

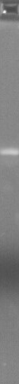

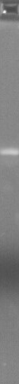

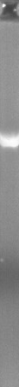

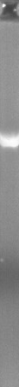

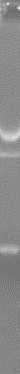

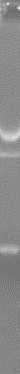

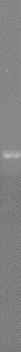

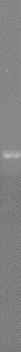

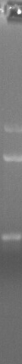

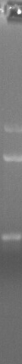

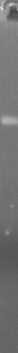

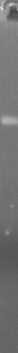

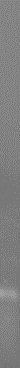

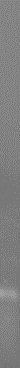

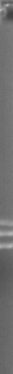

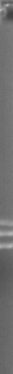

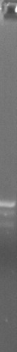

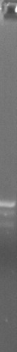

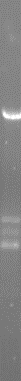

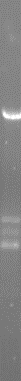

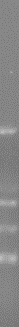

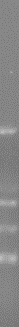

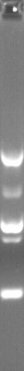

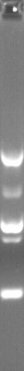

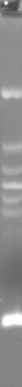

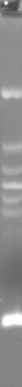

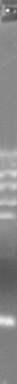

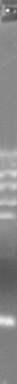

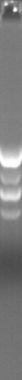

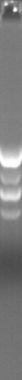

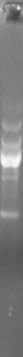

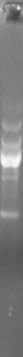

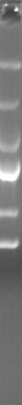


((((((Strain17,Strain68,Strain22,Strain71),Strain51,Strain103),(Strain10,Strain61,Strain4,Strain56)),(((((Strain30,Strain82,Strain31,Strain83),Strain34,Strain84),(Strain21,Strain72,Strain6,Strain60)),Strain27,Strain77),((((Strain50,Strain104,Strain52,Strain101),Strain53,Strain102),Strain12,Strain63),Strain5,Strain58))),Strain28,Strain79),(((((((((((Strain11,Strain64,Strain24,Strain76),Strain46,Strain98),Strain3,Strain57),Strain37,Strain89),(((Strain41,Strain92,Strain42,Strain93),Strain9,Strain62),(Strain39,Strain91,Strain40,Strain90))),Strain43,Strain95),Strain49,Strain99),(Strain33,Strain85,Strain7,Strain105)),(((Strain16,Strain106,Strain25,Strain78),(((((Strain13,Strain67,Strain2,Strain54),Strain26,Strain75),Strain48,Strain97),((Strain32,Strain80,Strain36,Strain86),Strain44,Strain94)),((Strain29,Strain81,Strain38,Strain88),Strain45,Strain96))),(Strain47,Strain100,Strain8,Strain59))),((((Strain1,Strain55,Strain14,Strain66),Strain20,Strain73),Strain35,Strain87),Strain23,Strain74)),(Strain18,Strain69,Strain19,Strain70))), (Strain15,Strain65).

Strains: 1, 2, 3, 4, 5, 11, 12, 13, 14, 15

Strains: 6-10

Strains: 16-29

Strains: 30-43

Strains: 44-53 (right to left)

Strains: 54-67

Strains: 68-81

Strains: 82-95

Strains: 96-106
